# Supplementary material for: Trend and predictive psychosocial factors of persistent depression/non-depression in Chinese adolescents: A three-year longitudinal study
Source: PLoS One. 2024 Oct 21;19(10):e0308303. doi: 10.1371/journal.pone.0308303 (PMC11493258; doi:10.1371/journal.pone.0308303)
Supplement: S1 Table — (DOCX) [file pone.0308303.s001.docx]

Trend and Predictive Psychosocial Factors of Persistent Depression/Non-depression in Chinese Adolescents: a Three-year Longitudinal Study

Short title: Persistent Depression/Non-depression in Adolescents

**Supplementary materials**

**S1 Table Comparison between dropouts and formal sample**

|  |  | | Drop-out (n = 243) | Final sample (n = 1301) | *F/x^2^* |
| --- | --- | --- | --- | --- | --- |
| Gender |  | |  |  | **2.438** |
| Male |  | | **139** | **666** |  |
| Female |  | | **104** | **621** |  |
| Missing data |  | |  | **14** |  |
| Siblings |  | |  |  | **3.074** |
| One Child |  | | **79** | **499** |  |
| Non-one child |  | | **164** | **799** |  |
| Missing data |  | |  | **3** |  |
| Migrant status | | |  |  | **2.692** |
| Migrant students |  | | **55** | **235** |  |
| Local students |  | | **188** | **1060** |  |
| Missing data | | |  | **6** |  |
| Positive Youth Development | | |  |  | **0.509** |
| Family intactness |  | |  |  | **1.444** |
| Intactness |  | | **232** | **1222** |  |
| Divorced parents |  | | **7** | **36** |  |
| Non-Intactness |  | | **2** | **24** |  |
| Others |  | | **2** | **8** |  |
| Missing data | | |  | **11** |  |
| Per capita monthly income in family (RMB) | | |  |  | **4.201** |
| ＜1,000 | |  | **3** | **27** |  |
| 1,000-1,999 | |  | **21** | **81** |  |
| 2,000-2,999 | |  | **25** | **135** |  |
| 3,000-3,999 | |  | **41** | **169** |  |
| 4,000-4,999 | |  | **24** | **121** |  |
| 5,000-5,999 | |  | **33** | **127** |  |
| ≥6,000 | |  | **88** | **439** |  |
| Missing data | |  | **8** | **202** |  |
| Father’s Education Level | |  |  |  | **3.814** |
| Middle school or lower | |  | **84** | **404** |  |
| High school or college | |  | **101** | **451** |  |
| Graduate | |  | **41** | **226** |  |
| Above graduate | |  | **16** | **120** |  |
| Minssing data | |  | **1** | **100** |  |
| Mother’s Education Level | |  |  |  | **1.437** |
| Middle school or lower | |  | **99** | **492** |  |
| High school or college | |  | **90** | **427** |  |
| Graduate | |  | **36** | **217** |  |
| Above graduate | |  | **13** | **74** |  |
| Missing data | |  | **5** | **91** |  |
