# Supplementary material for: Trend and predictive psychosocial factors of persistent depression/non-depression in Chinese adolescents: A three-year longitudinal study
Source: PLoS One. 2024 Oct 21;19(10):e0308303. doi: 10.1371/journal.pone.0308303 (PMC11493258; doi:10.1371/journal.pone.0308303)
Supplement: Attachment — Submitted filename: Recommendations research review.docx [file pone.0308303.s003.docx]

# Recommendations:

While the introduction highlights the importance of studying non-depressive symptoms, it could further elaborate on why this focus is unique and how it can contribute to the field of adolescent mental health

The ecological system theory is mentioned as a framework, but there is little discussion on how this theory has been applied in previous research on adolescent depression.

A theoretical framework could have been drawn as a support/guidance

While the attrition rate of 15.7% is mentioned, there is no detailed analysis of the characteristics of participants who dropped out compared to those who remained. This could help understand if attrition biased the results.

Suggestion: Include a comparison of demographic and baseline characteristics between completers and dropouts to assess potential attrition bias.

The sample could have been drawn from schools of various socioecnomical status to increase the generalizability of the results

Seasonal effect on the depression (winter depression) could have been mentioned as it can act as a confounding factor.

Elaborate on how ecological system theory informed the study design, variable selection, and the interpretation of findings.
